# Evaluating the Impact of Little Cigar Use on the Oral Bacterial Microbiota of Cigarette Smokers

**DOI:** 10.3390/pathogens15070732

**Published:** 2026-07-13

**Authors:** Suhana Chattopadhyay, Leena Malayil, Emmanuel F. Mongodin, Amy R. Sapkota

**Affiliations:** 1 Department of Global, Environmental, and Occupational Health, University of Maryland School of Public Health, College Park, MD 20742, USA; suhanac@umd.edu (S.C.); lmalayil@umd.edu (L.M.); 2 Institute for Genome Sciences, University of Maryland School of Medicine, Baltimore, MD 21201, USA; emmanuel.mongodin@nih.gov

**Keywords:** little cigars, smoking, oral microbiome, bacteria, pathogens

## Abstract

Tobacco products (e.g., cigarettes, little cigars) harbor diverse bacterial communities and long-term tobacco use alters the oral microbiome, potentially leading to oral disease. However, no studies have evaluated the immediate changes in the oral bacterial microbiota that could occur after using a new tobacco product. To address this knowledge gap, buccal swab and saliva samples were collected from forty cigarette smokers before and after a single use of a little cigar product on two separate visits. Total DNA was extracted from a total of 320 samples. The 16S rRNA gene was amplified from these samples and sequenced on the Illumina HiSeq to characterize the bacterial microbiota. Oral bacterial diversity was not significantly different between pre- and post-smoking samples. However, post-smoking buccal samples were enriched with *Delftia*, *Leptotrichia*, *Pseudomonas* and *Stenotrophomonas* (genera that can include opportunistic pathogens) and post-smoking saliva samples were enriched with *Catonella* when compared to the pre-smoking samples. In summary, single use of a little cigar product does not immediately impact overall oral bacterial diversity among cigarette smokers; however, post-smoking oral samples may have a higher relative abundance of some bacterial genera. Hence, smoking a new tobacco product could potentially alter the relative abundance of some bacterial types within the oral cavity of smokers, highlighting a potential microbiological pathway through which tobacco use could contribute to oral disease risk.

## 1. Introduction

Tobacco products have been shown to harbor a myriad of bacterial communities [1,2,3,4,5,6]. A recent study from our group also demonstrated that viable bacterial genera (e.g., *Bacillus*, *Paenibacillus* and *Terribacillus*) originating from cigarette tobacco can be aerosolized in mainstream cigarette smoke [7]. The oral cavity is the first to encounter this mainstream smoke and hence has the greatest potential to be affected by it. While the long term use of tobacco products has been well established to cause dysbiosis (community disturbance) in the oral microbiome of users [8,9,10,11,12], the potential transfer of tobacco-related bacteria to the oral cavity or the immediate impacts of using a specific tobacco product on a user’s oral bacterial microbiota have not been evaluated. This is of significant interest, since changes in bacterial diversity and community composition of the oral cavity can potentially initiate inflammation and subsequent disease development, including the growth of malignant lesions in the mouth [13,14,15].

These types of changes in the mouth can occur in users of a diverse range of tobacco products, from cigarettes to little cigars. Little cigars are comparable to traditional cigarettes in size and shape but contain more tobacco (100–200 mg) and other chemical compounds [16,17]. In comparison to cigarettes, little cigars also have a greater puff volume and puff duration, delivering more carbon monoxide, tobacco-specific nitrosamines (TSNAs), and benzo(a)pyrene to users than cigarettes [17,18,19], consequently causing greater cytotoxicity and inflammation [16]. Similarly to cigarettes, the tobacco in little cigars has been shown to harbor diverse bacterial communities [5,20]. For example, Smyth et al. (2019) demonstrated that the predominant bacterial genera within little cigar tobacco are *Pantoea*, *Pseudomonas* and *Staphylococcus*, including multiple bacterial pathogens such as *Staphylococcus sciuri* and *Pseudomonas pseudoalcaligenes* [5]. Nevertheless, to our knowledge there are no data concerning whether or not the bacterial communities within little cigars could be transferred to the oral cavity of smokers and effect changes in the oral microbiome.

Oral microbiome dysbiosis from extrinsic perturbations such as tobacco smoking is dependent on the amount and frequency of products smoked/used per day [21]; however, the overall composition of the oral microbiome has been shown to remain relatively stable over time [22,23,24]. Yet, to date, studies evaluating temporal changes in the oral microbiome [25,26,27,28] have not employed the use of tobacco products by study subjects, and hence, data on potential transient oral microbiome shifts among smokers are limited.

Other studies characterizing oral microbiome dysbiosis associated with tobacco smoking have focused on the use of traditional and/or electronic cigarettes [12,21,25,29,30,31] and indicated that specific oral bacterial genera are impacted by exposure to smoke over a one-week period [8]. For example, greater shifts in bacterial communities among smokers’ plaque samples were demonstrated compared to those of non-smokers over 7 days, with the enrichment of a pathogen-dominated bacterial community (e.g., *Fusobacterium nucleatum*, *Acinetobacter johnsonii*, *A. baumannii*, *Streptococcus mutans*) among smokers [8]. Moreover, while levels of predominant genera such as *Streptococcus*, *Neisseria*, and *Veillonella* were stable over seven days among non-smokers, less stability was observed among smokers during the same time period, while the colonization of pathogens associated with periodontitis occurred within 24 h of biofilm development [8]. However, to our knowledge, no study has evaluated the immediate potential changes in oral bacterial communities after a single use of a little cigar. Defining immediate changes in the oral microbiota is clinically important because even short-term shifts toward opportunistic pathogens may represent an early mechanistic step in tobacco-related oral disease development. Understanding these impacts can also inform risk assessment, product regulation, and targeted messaging around the harms of emerging tobacco products. Therefore, the objective of our study was to evaluate transient changes in the oral bacterial microbiota after cigarette users smoked a single little cigar product on two different occasions.

## 2. Methods

### 2.1. Product Selection

Swisher Sweets little cigars were chosen for inclusion in the study because they are characterized by the highest market sales among little cigar brands in the U.S. [32]. Two products (Swisher Sweets Original (SSORG) and Swisher Sweets Cherry (SSCHR)) were purchased in Columbus, OH, USA, and stored at 4 °C (to minimize variability and maintain consistent conditions prior to use) in their original packaging before being smoked by study participants.

### 2.2. Sample Size Calculation

Data from the Human Microbiome Project (HMP) Consortium, as detailed in a previously published article [33], provides a resource for power analysis by empirically quantifying the variability of the healthy oral microbiome. The HMP obtained taxonomic profiles from 233 oral 16S rRNA datasets (131 buccal, 102 saliva) and examined genus-level variability across the sampled population. They identified 30 genus-level groups (14 in buccal, 16 in saliva) that accounted for at least 1% of 16S rRNA sequences in the average oral microbiome and measured the variance in relative abundance for each genus. The power analysis conducted estimated the power for detecting various shifts in average relative abundance, with increased power found in higher-abundance genera. For this study, focusing on genera representing 1–3% of 16S rRNA sequences and requiring a significance level of α = 0.05, the analysis suggested that a sample size of at least 20 subjects per trial would provide robust statistical power to detect small percentage changes in low-abundance taxa. To account for potential loss to follow-up, we planned to over-recruit by 20%, which would result in an anticipated study population of 24 subjects per trial.

### 2.3. Study Population

Participants were recruited through word-of-mouth in Columbus, OH, USA. Once an individual expressed interest in participating in the study, they completed a phone screening process to ensure that they met our inclusion criteria. All participants had to be healthy individuals who self-recognized as current smokers (smoked at least six tobacco products on a typical day for the previous three years). Inclusion criteria included generally good oral health, with no antibiotic use, no heart or lung problems and no diagnosis of pneumonia in the past six months. Exclusion criteria included untreated lesions or oral abscesses in the mouth, clinically diagnosed candidiasis or halitosis, a pregnancy or plans to become pregnant in the next six months. The study was approved by the Battelle, OH, Institutional Review Board and all research was conducted in accordance with the Declaration of Helsinki. All participants completed the informed consent process and signed consent forms on their first visit.

### 2.4. Laboratory Visits, Smoking Process, and Sample Collection

Upon recruitment, study participants visited the laboratory twice, with 24 h to 35 days between visits. During their first visit, participants completed three questionnaires focused on demographics, tobacco product use, and oral health history. During each of the two visits, each participant provided pre-smoking (PRE) buccal swab and saliva samples then smoked one of two tobacco products (SSCHR on visit 1 or SSORG on visit 2), and then provided post-smoking (POST) buccal swab and saliva samples. For the purpose of this study, the term ‘new’ tobacco product refers to the little cigar product, which was ‘new’ to the participants, all of whom self-identified as traditional cigarette smokers.

For saliva samples, participants were asked to let saliva form in their mouth for at least one minute before collecting 2–5 mL in a 50 mL falcon tube. RNALater (3x volume) solution (Thermo Fisher, Waltham, MA, USA) was then added to the saliva sample, and the sample was vortexed and then incubated at 4 °C for 24 h. After incubation, all samples were frozen at −80 °C until DNA extractions could be completed. Buccal swab samples were collected with four e-swabs (Copan Diagnostics, Murrieta, CA, USA) from four oral sites: the tongue dorsum, the hard palate, and the left and right buccal mucosa. Surfaces were swabbed with e-swabs for 1 min, and all four e-swabs were added to a single 50 mL Falcon tube with 5 mL RNALater solution. Similarly to the saliva samples, all of the buccal swab samples were vortexed and incubated at 4 °C for 24 h. Afterwards, samples were then frozen at −80 °C until DNA extractions could be completed.

### 2.5. Total DNA Extraction, 16S rRNA Gene Amplification, and Sequencing

All buccal swab and saliva samples were thawed on ice. To 500 µL of each saliva sample, 500 µL of ice-cold 1× molecular-grade Phosphate-Buffered Solution (PBS) was added. Both sample types (buccal swabs and saliva) were then centrifuged at 10,000 rpm for 30 min. Next, the supernatant was discarded and the pellet was resuspended in 1 mL of ice-cold 1× PBS, and then transferred into Lysing Matrix B tubes (MP Biomedicals, Solon, OH, USA). DNA extraction was carried out following previously published protocols using enzymatic digestion and mechanical lysis of cells [34]. Briefly, samples were incubated twice in water baths with the addition of two enzymatic cocktails (Cocktail A: Mutanolysin, Lysostaphin, and Lysozyme; Cocktail B: Proteinase K and 10% sodium dodecyl sulfate). After mechanical lysis of cells using an MP Biomedical FastPrep 24 (Santa Ana, CA, USA), DNA lysate was purified using a QIAmp DSP DNA mini kit (Qiagen, Germantown, MD, USA) according to the manufacturer’s protocol. PCR amplification of the V3V4 hypervariable region of the 16S rRNA gene was then carried out using 319F (ACTCCTACGGGAGGCAGCAG) and 806R (GGACTACHVGGGTWTCTAAT) universal primers. Each primer was barcoded with a linker sequence and a 12 bp heterogeneity spacer index sequence. Amplicons were purified using the SequelPrep Normalization Kit (Invitrogen Inc. Carlsbad, CA, USA). Samples were pooled at a final concentration of 25 ng/amplicon and the pooled samples were sequenced on an Illumina HiSeq2500 (Illumina, San Diego, CA, USA), using previously published protocols [35,36].

### 2.6. Sequence Quality Filtering and Bioinformatic Analysis

16S rRNA sequencing reads were screened for low quality and short length, assembled using PANDAseq, Version 2.11, demultiplexed, and chimera-trimmed using UCHIME (v. 4). Quality reads were then incorporated into Quantitative Insights Into Microbial Ecology (QIIME v1.9.0) and clustered de novo using VSEARCH (v. 2.31.0). Taxonomies were then assigned using the Greengenes database (v. 132), using a 0.97 confidence threshold. The resulting operational taxonomic unit (OTU) table, reference sequences, and phylogenetic tree files were imported into R Statistical computing software (v. 0.99.473) using the phyloseq R package (1.22.3) for downstream analysis.

The Phyloseq package [37] in R was used to calculate alpha diversity and the results were tested for significance using ANOVA. Cumulative sum scaling (CSS) was used to normalize reads using the MetagenomeSeq (v. 1.16.0) package [38], and normalized reads were used to compute beta diversity using the vegan (v. 2.7-2) and Phyloseq (v. 1.22.3) packages in R and the results were tested for significance using ANOSIM. Core bacterial microbiota profiling was performed at the genera level when the genus was prevalent in at least 20% of the samples at a relative abundance of 0.2 on Microbiome Analyst [39,40]. Decision trees were generated using the Random Forest algorithm to predict bacterial biomarker taxa associated with each time point (PRE and POST) for each sample type (buccal swabs and saliva).

## 3. Results

### 3.1. Study Participants

We recruited a total of 40 participants, exceeding the target sample size of 24 by 67%, thereby increasing the statistical power of the study. Of these, 40% identified as female and 47.5% identified as Black (Table 1). The majority (55%) of the participants were single (or never married) and 58% were between 25 and 45 years old. All of the participants were current cigarette smokers: 35.13% smoked Newport brands and 21.6% smoked Marlboro products, with the majority (59.4%) smoking mentholated varieties of the cigarette brand that they used. Over 90% of the participants had previously smoked a little cigar product, with 23.5% currently smoking a little cigar every day and 53% smoking little cigars on some days (Table 2). Thirty-two percent of the subjects who had used little cigars had smoked Swisher products, followed by 20.5% who had used a Black & Mild product. Over 60% of the subjects smoked a flavored product.

### 3.2. Sequencing Data

A total of 320 samples yielded 2,472,070 sequences, comprising 713 OTUs, with an average number of sequences per sample of 11,392.03 (±8563.63 SD). After removing low-quality samples and samples with Good’s coverage values ≤ 0.95, there were 2,469,617 sequences from 208 samples, with an average number of sequences per sample of 11,873.16 (±8421.07 SD), which were used for downstream analysis.

### 3.3. Transient Changes in Oral Bacterial Diversity 

**Figure 1:**
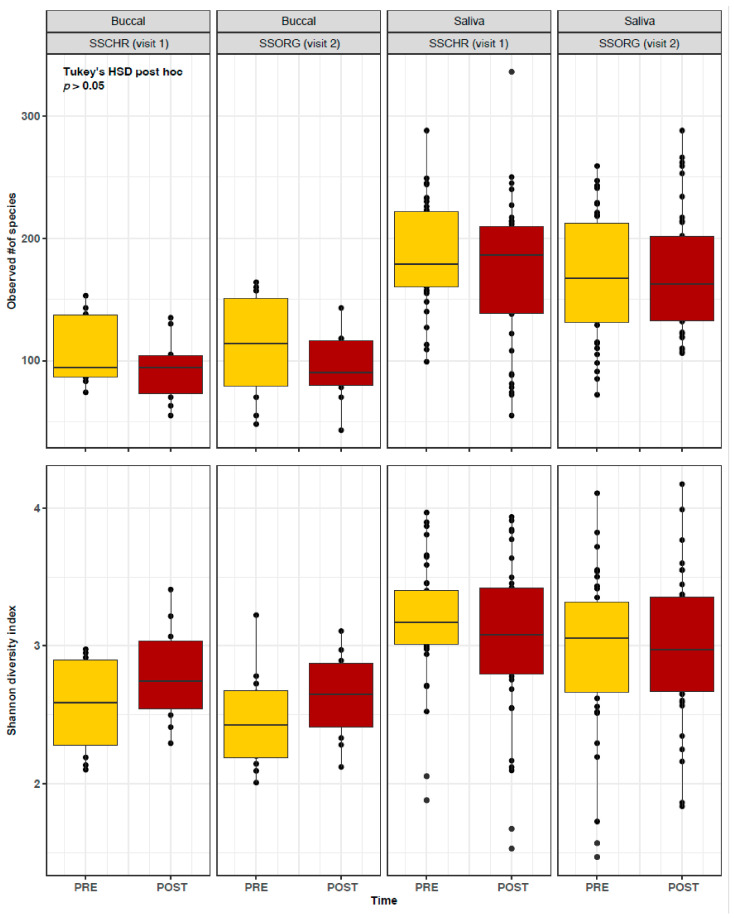
Alpha diversity analysis of buccal swab and saliva samples collected pre- and post-smoking of two little cigar products: Swisher sweets original (SSORG) and Swisher sweets cherry (SSCHR). The pre-smoking samples are denoted with yellow bars and post-smoking samples are denoted with red bars. Diversity was measured between pre-smoking (PRE) and post-smoking (POST) samples using ANOVA with Tukey’s HSD post hoc test.

Alpha diversity was measured using the observed number of species metric and the Shannon index (Figure 1). There were no significant differences in alpha diversity (*p* > 0.05) between PRE and POST smoking samples for each of the tobacco products stratified by sample type (buccal swab and saliva) across all study participants. When we looked at participant-level alpha diversity, there were also no significant differences between PRE and POST samples for each participant during both visits (Figure S1).

**Figure 2:**
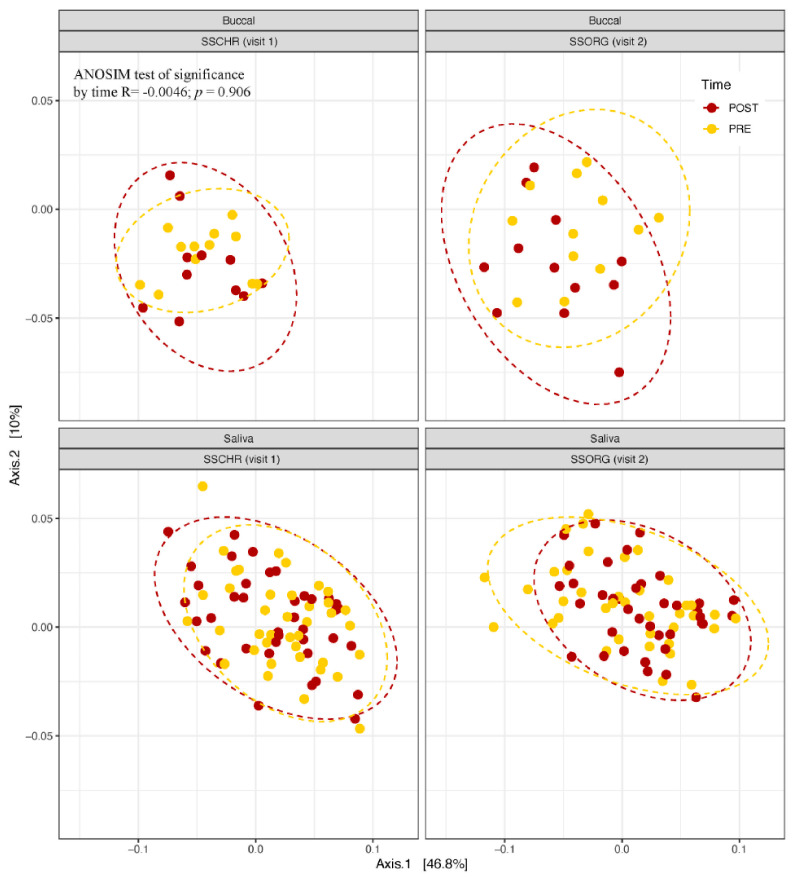
PCoA plot measuring beta diversity between pre- and post-smoking samples from each visit. Ellipses are drawn at 95% confidence intervals. The statistical significance of beta diversity by time was measured by the ANOSIM test of significance and *p*-values < 0.05 were considered significant.

To evaluate beta diversity between time points (PRE and POST), we performed Principal Coordinates Analysis (PCoA) on weighted UniFrac distance matrices of normalized OTU relative abundances. (Figure 2). The first two principal axes explained 56.8% of the variation in bacterial community composition. Similarly to the alpha diversity results, there were no statistically significant changes (ANOSIM *p* > 0.05) in bacterial diversity between PRE and POST buccal swab samples or PRE and POST saliva samples for both visits. Performing further evaluation at the participant level, we also compared the UniFrac distances for each subject for each sample. PRE and POST smoking samples (of each sample type) from a single participant were characterized by the lowest UniFrac distances and clustered closer together when compared to samples from other participants.

### 3.4. Core Bacterial Microbiota and Correlation Between Bacterial Genera in Pre-Smoking Samples

The core oral bacterial microbiota in buccal swab and saliva samples from PRE smoking samples was significantly dominated by five taxa: *Streptococcus*, *Veillonella*, *Rothia*, *Actinomyces*, *Granulicatella*, *Haemophilus* and *Prevotella* (Figure S2). Among all bacterial genera, *Streptococcus* was at the highest relative abundance in both buccal swab (49%) and saliva (38.2%) samples. While *Rothia* (16%) and *Veillonella* (12%) were the other two bacterial genera at >10% relative abundance in buccal swabs, *Veillonella* was at a 14.2% relative abundance in saliva samples. Other genera identified in both buccal swab and saliva samples included *Lactobacillus* (1.5% buccal swabs and 2.2% in saliva), *Pseudomonas* (1.2% in buccal swabs and 2.4% in saliva), *Stenotrophomonas* (1% in buccal swabs) and *Atopobium* (1.3% in saliva).

### 3.5. Bacterial Taxa Associated with a Single Smoking Exposure 

**Figure 3:**
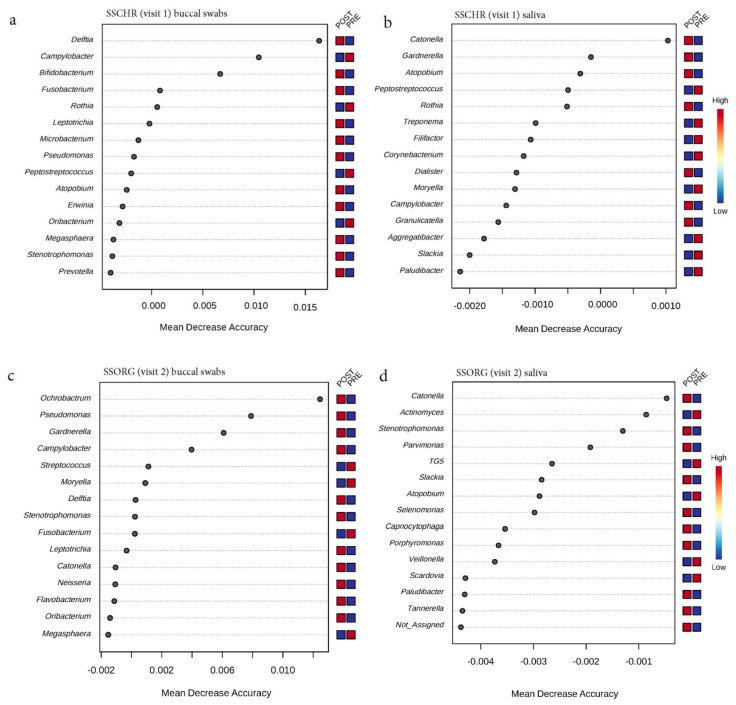
Predicted association of bacterial species between pre- (PRE) and post-smoking (POST) samples using the Random Forest algorithm in (**a**) buccal swabs from visit 1; (**b**) saliva from visit 1; (**c**) buccal swabs from visit 2; and (**d**) saliva from visit 2. The increasing abundance of each bacterial genera is represented by the color scale (blue to red).

A Random Forest algorithm was applied to construct decision trees to predict the bacterial taxa best associated with PRE and POST samples for both buccal swab and saliva samples (Figure 3). The POST buccal swab samples from both visits were enriched with four bacterial genera, *Delfia*, *Leptotrichia*, *Pseudomonas*, and *Stenotrophomonas*, when compared to the PRE samples (Figure 3a,c). The POST saliva samples were enriched with *Catonella* when compared to the PRE samples from both visits (Figure 3b,d). After a single exposure to SSCHR, both buccal swab and saliva POST samples were associated with a depletion of *Peptostreptococcus*, with enrichment of *Atopobium* (Figure 3a,b). After a single use of an SSORG product, the higher relative abundance of *Stenotrophomonas* and *Catonella* were associated with POST samples, compared to PRE samples for both buccal swab and saliva samples (Figure 3c,d).

### 3.6. Changes in the Relative Abundance of Bacterial Taxa with a Single Exposure to Smoking

**Figure 4:**
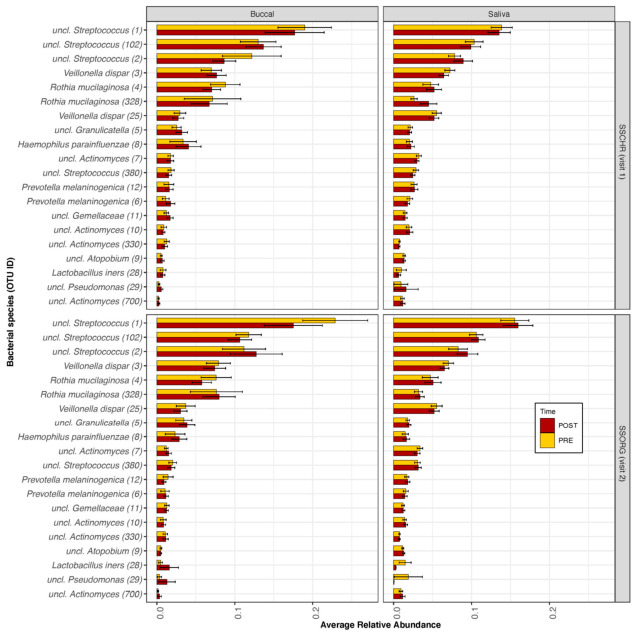
Average relative abundance (±SE) of the top 20 bacterial species (OTU ID) present in buccal swab and saliva samples from pre- (denoted in yellow bars) and post-smoking (denoted in red bars) samples in each visit.

To evaluate the transient changes in the abundance of specific bacterial taxa, we compared the mean relative abundance of the top 20 bacterial OTUs between the PRE and POST smoking samples across buccal swab and saliva samples separately (Figure 4). Three OTUs among the *Streptococcus* genera (OTU # 1, 102, and 2), *Veillonella dispar* (OTU# 3 and 25), *Rothia mucilaginosa* (OTU # 4 and 328), and *Prevotella melaninogenica* (OTU# 12 and 6) were identified among all samples. There were no statistically significant differences in the relative abundance of the top 20 bacterial taxa between PRE and POST samples when stratified by product smoked and sample type.

### 3.7. Differences in the Oral Bacterial Microbiota Within and Between Participants

To evaluate changes in the oral bacterial microbiota within participants (intra-individual variation), we compared the PRE samples per participant across sample types and time points (visits). There were no significant differences in bacterial community composition between PRE and POST buccal swab and saliva samples across the two time points for a single individual participant (Table 3). However, the UniFrac distances between bacterial community members within an individual were lower in the buccal swab samples compared to the saliva samples.

**Figure 5:**
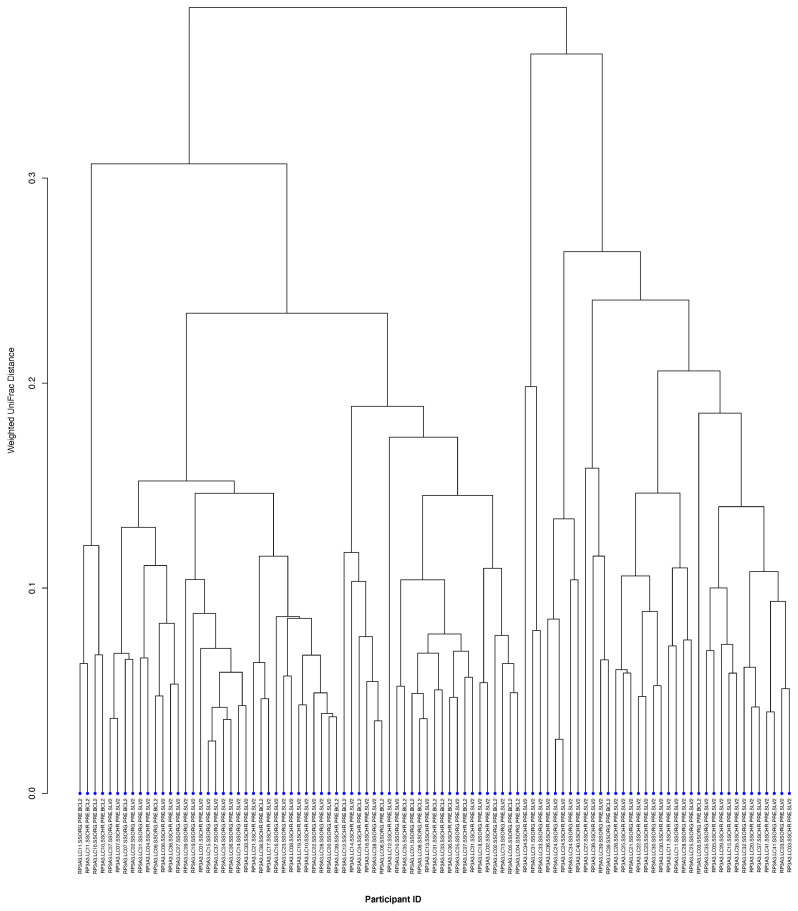
Histogram of weighted UniFrac distances of all pre-smoking samples across all participants.

To evaluate bacterial diversity differences between individuals (inter-individual variation), we compared the PRE smoking samples from all 40 participants at each time point. Shannon diversity metrics among all participants were characterized by statistically significant inter-individual differences, among both buccal swab (*p* = 0.0195) and saliva samples (*p* = 0.0004) (Figure S1). Beta diversity was evaluated using weighted UniFrac distances between all pre-smoking samples (Figure 5). Across both sample types, while there were no significant differences on their first visit (buccal swabs ANOSIM R = −0.0799; *p* > 0.05; saliva ANOSIM R = 0.724; *p* > 0.05), there was significant variation in bacterial community composition across participants during their second visit (buccal swab ANOSIM R = 0.5004; *p* < 0.05; saliva ANOSIM R = 0.5003; *p* < 0.05). The inter-individual variation in bacterial genera was also different among the top ten most abundant bacterial genera present in the PRE smoking samples across all participants (Table S1).

## 4. Discussion

This study evaluated whether or not immediate changes in oral bacterial microbiota occurred after cigarette smokers used a new tobacco product. Overall, our data show that the oral bacterial communities of cigarette smokers remain stable after exposure to the mainstream smoke of a single little cigar product. Furthermore, our data provide a comprehensive characterization, as well as further evidence of the temporal stability, of the oral bacterial microbiota of cigarette smokers.

Previous studies from our group that have evaluated bacterial communities present in the tobacco of little cigars have identified multiple bacterial genera (e.g., *Staphylococcus*, *Pseudomonas*, *Corynebacterium* and *Bacillus*) in Swisher Sweets little cigars [5,20]. In the present study, the bacterial genera that were associated with our post-smoking samples were identified as *Delftia*, *Leptotrichia*, *Pseudomonas*, *Stenotrophomonas* and *Catonella*. While *Pseudomonas* has been identified in little cigar tobacco products, the other above-mentioned bacterial genera have not been identified in these products. Previous studies have demonstrated that the number of cigarettes smoked and duration of cigarette smoking influence the composition of periodontal pathogens found in smokers’ oral cavities [41,42]. Smokers have also been shown to exhibit relatively unstable biofilm occurrence (when compared to non-smokers) at least within 24 h, including early colonization of periodontal pathogens (e.g., *Fusobacterium*, *Cardiobacterium* and *Selenomonas*) [8]. Yet, in the present study, either the single exposure to little cigar smoke might not have been adequate to trigger these types of responses in smokers, or our methods were not sensitive enough to detect these changes.

Looking into specific bacterial taxa, we found that *Streptococcus* was the most abundant genera among all participants, followed by *Rothia, Veillonella*, and *Actinomyces*. While the relative abundance of *Streptococcus*, *Veillonella* and *Actinomyces* was lower in post-smoking samples (when compared to pre-smoking samples) at both visits, *Rothia* decreased in relative abundance only on visit two. While a number of studies have pointed out the role of commensals in oral disease development [43,44], the contribution of specific putative pathogens in oral disease initiation is still unclear [45]. In contrast, a higher relative abundance of all of these genera has been shown previously in healthy smokers [46], defining the “core microbiome” of the oral community [47].

While we observed the above-mentioned bacterial genera in all of our samples in comparatively higher relative abundance, we also identified a few genera (that could potentially include opportunistic pathogens) associated with the post-smoking samples (e.g., *Delftia*, *Leptotrichia*, *Stenotrophomonas*, *Pseudomonas* and *Atopobium*). Demonstrating resistance to multiple groups of antibiotics, ubiquitous *Delftia* is an emerging member of the opportunistic healthcare-associated pathogens [48,49,50], a few species of which are also used as plant growth-promoters [51]. Another common oral cavity member, *Leptotrichia*, has been frequently implicated in periodontal diseases and tooth decay [52]. With the production of potent endotoxin, *Leptotrichia* has been associated with a spectrum of human diseases [52,53]. *Stenotrophomonas* has been noted as a persistent colonizer of biofilms, but antibiotic resistance renders some species of this genus as major players in the development of infectious diseases, specifically among immunocompetent individuals [54,55]. Finally, *Atopobium* has been associated with periodontal diseases and gingival squamous cell carcinomas [56,57].

In addition to describing bacterial genera that were identified in the smokers’ mouths, we also compared variations within bacterial community composition (1) across all subjects at one visit (inter-individual variation) and (2) within each subject across two visits (intra-individual variation). Comparing across individuals, we found significantly more variation in the oral bacterial microbiota in comparison to intra-individual variation. This was consistent with previous studies that demonstrated that oral bacterial community composition is variable among participants, given the multiple intrinsic and extrinsic factors that can affect the oral microbiome of an individual [27,58,59,60]. We also found that the degree of temporal variability (measured with the Shannon diversity index per individual) in the composition of oral bacterial communities was different across all individuals. This was consistent with previous microbiome studies of samples from other areas of the body such as the palm, gut, tongue and vagina [58,61]. In our study, although there was considerable variation between subjects, bacterial profiles within subjects (intra-individual) were stable over the study period. Previous studies have shown similar results when evaluating a range of time periods (24 h to 10 months) [59,60].

There are multiple strengths to note in this study. To our knowledge, this is the first study to investigate potential transient changes in the oral bacterial microbiota resulting from a single exposure to a little cigar. While previous studies have evaluated oral microbiome impacts from tobacco smoking [11,62,63,64], none have sought to assess the immediate impacts on the oral bacteria of the smoker after a single use of a product. In addition, our data relied on high-throughput 16S rRNA sequencing from a robust sample size, and sampling from two different oral cavity locations.

Nevertheless, there were notable limitations to this study as well. First, we could not measure all of the possible factors that could contribute to individual-level variations in the oral bacterial microbiota. Possible factors driving such variations could be an individual’s genetic makeup, diet, or lifestyle/behavior [65,66]. Another limitation of our study was the inability to assess functional capabilities of the oral bacterial microbiota, since we were not able to subject our samples to a metagenomic sequencing approach due to costs. As a result, our analysis is limited to taxonomic compositions inferred from 16S rRNA gene sequencing, and we are unable to directly evaluate microbial gene content or metabolic potential that may underlie the observed community shifts. Additionally, since our work only included data from 16S rRNA gene sequencing, this limited our ability to complete species-level taxonomic assignments and prevented us from determining whether or not detected bacterial genera were viable/live or associated with relic DNA.

## 5. Conclusions

In conclusion, we found that a single use of a little cigar product did not immediately impact overall oral bacterial diversity among cigarette smokers. However, some bacterial genera were found to be associated with post-smoking samples (*Delftia*, *Leptotrichia*, *Pseudomonas*, *Stenotrophomonas* and *Catonella*). Future studies, utilizing a combination of alternative methods (e.g., culture methods, qPCR, metagenomic sequencing), would be needed to evaluate if those bacteria detected in post-smoking oral samples originated from the little cigar products/mainstream smoke and were transferred to the oral cavity, or if they were already present in the oral cavity and were potentially enriched after exposure to mainstream smoke.

## Figures and Tables

**Table 1:** Demographics of the study participants.

	n = 40	%
Gender		
Female	16	40
Male	21	52.5
Don’t know/Refused	3	7.5
Age		
25–35	11	27.5
36–45	11	27.5
46–55	8	20
55+	7	17.5
Don’t know/Refused	3	7.5
Marital Status		
Legally married	2	5
Living with Partner	4	10
Single/never married	22	55
Divorced	7	17.5
Separated	2	5
Don’t know/Refused	3	7.5
Race		
Black or African American	19	47.5
White	18	45
Don’t know/Refused	3	7.5
Employment		
Full-Time, 35+ h/week	13	32.5
Part-Time, irregular h/day work	7	17.5
Part-Time, regular h	5	12.5
Retired/Disabled	1	2.5
Homemaker	2	5
Unemployed	9	22.5
Don’t know/Refused	3	7.5

**Table 2:** Little cigar use of all study subjects. For frequency of smoking little cigars, ‘Everyday’ refers to smokers who used little cigars on all days of the past 30 days, ‘Some days’ refers to smokers who used little cigars only on few days in the past 30 days, and ‘Not at all’ refers to smokers who did not smoke a little cigar in the past 30 days.

Subject ID	Tried Little Cigar	Little Cigar Use		
		Frequency of Smoking	Brand	Flavor
LC01	Yes	Some days	Swisher Sweets	Full Flavor
LC02	Yes	Some days	Don’t know	Menthol
LC03	Yes	Not at all	Winchester	Regular
LC04	Yes	Everyday	Garcia game	Grape/honey
LC05	Yes	Not at all	Black & Mild	Regular
LC06	Yes	Not at all	Swisher	Grape
LC07	Yes	Everyday	Black & Mild	Regular
LC08	Yes	Not at all	NA	NA
LC09	Yes	Some days	Swisher Sweets	Regular, grape, sour apple
LC10	Yes	Some days	Swisher	
LC11	Yes	Everyday	Seneca	Full Flavor
LC12	Yes	Some days	Swisher Sweets	Regular
LC13	Yes	Some days	Swisher	Regular
LC14	Yes	Some days	Garcia vega game and Swisher Sweets	Cherry
LC15	Yes	Everyday	Black & Mild	Cigarillo
LC16	Yes	Not at all	NA	NA
LC17	Yes	Everyday	Swisher and Black & Mild	Tropical
LC18	No	Not at all	NA	NA
LC19	Yes	Some days	Cigarillos	Cherry
LC20	Yes	Some days	Black & Mild	Wine
LC21	Yes	Not at all	Swisher	Regular
LC22	Yes	Everyday	Swisher Sweets	Grape
LC23	Yes	Not at all	Swisher	Regular
LC24	Yes	Some days	Black & Mild	Regular
LC25	Yes	Some days	Swisher Sweet Cigarillos	Plain
LC27	No	Not at all	NA	NA
LC28	Yes	Everyday	Cheyenne	Full flavor
LC29	Yes	Some days	Swisher Sweet Cigarillos	Peach
LC30	Yes	Everyday	Swisher Sweet Cigarillos	Sweet
LC31	No	Not at all	NA	NA
LC32	Yes	Some days	Seneca	Regular
LC33	Yes	Not at all	NA	NA
LC34	Yes	Some days	show	Mango
LC35	Yes	Some days	Black & Mild	Wine
LC36	Yes	Some days	Djarum	Clove
LC37	Yes	Some days	Swisher	Regular
LC38	Yes	Some days	Black & Mild	Cherry blend
LC39	Yes	Not at all	NA	NA
LC40	Yes	Some days	Any/Random	Cherry blend
LC41	Yes	Some days	Black & Mild	Regular

**Table 3:** Within and between individual variation in UniFrac distances.

Variation	Sample Type	Product Smoked	Time	UniFrac Distance	
		Visit #		Average	SD
Intra-individual (within a subject)	Saliva	Visits 1 + 2	Pre	0.11	0.07
			Post	0.11	0.08
	Buccal	Visits 1 + 2	Pre	0.09	0.04
			Post	0.11	0.06
Inter-individual (between subjects)	Saliva	Visits 1 + 2	Pre	0.16	0.07
			Post	0.18	0.08
		SSCHR (visit 1)	Pre	0.16	0.06
			Post	0.17	0.07
		SSORG (visit 2)	Pre	0.17	0.08
			Post	0.18	0.09
	Buccal	Visits 1 + 2	Pre	0.15	0.06
			Post	0.15	0.06
		SSCHR (visit 1)	Pre	0.14	0.06
			Post	0.14	0.05
		SSORG (visit 2)	Pre	0.17	0.06
			Post	0.16	0.07

## Data Availability

Data concerning the samples included in this study are deposited under the NCBI BioProject accession number PRJNA690810.

## Supplementary Materials

The following supporting information can be downloaded at https://www.mdpi.com/article/10.3390/pathogens15070732/s1. Figure S1: Alpha diversity of oral bacterial microbiota per individual subject from buccal and saliva samples collected pre- (red) and post-smoking (yellow) of two separate little cigar products: Swisher sweets original (SSORG) and Swisher sweets cherry (SSCHR); Figure S2: Core bacterial genera present in (a) buccal swab samples and (b) saliva samples. The color gradient shows the prevalence (%) of each genus from zero (blue) to all (red) samples; Table S1: Relative abundance of top 10 bacterial genera in pre-smoking samples across all participants during two visits.
