# Supplementary material for: Evaluating the Impact of Little Cigar Use on the Oral Bacterial Microbiota of Cigarette Smokers
Source: Pathogens. 2026 Jul 13;15(7):732. doi: 10.3390/pathogens15070732 (PMC13415183; doi:10.3390/pathogens15070732)
Supplement: Supplementary file 1 [file pathogens-15-00732-s001.zip › pathogens-4391634-updated supplementary_071426.pdf]

Article

Evaluating the Impact of Little Cigar Use on the Oral Bacterial Microbiota of Cigarette Smokers

Suhana Chattopadhyay [1], Leena Malayil [1], Emmanuel F. Mongodin [2,†] and Amy R. Sapkota [1,*]

1 Department of Global, Environmental, and Occupational Health, University of Maryland School of Public Health, College Park, MD 20742, USA; suhanac@umd.edu (S.C.); lmalayil@umd.edu (L.M.)
2 Institute for Genome Sciences, University of Maryland School of Medicine, Baltimore, MD 21201, USA; emmanuel.mongodin@nih.gov
* Correspondence: ars@umd.edu
† Current address: Division of Lung Diseases, National Heart, Lung and Blood Institute (NHLBI),
National Institutes of Health (NIH), Bethesda, MD 20892, USA

Supplementary Figures:

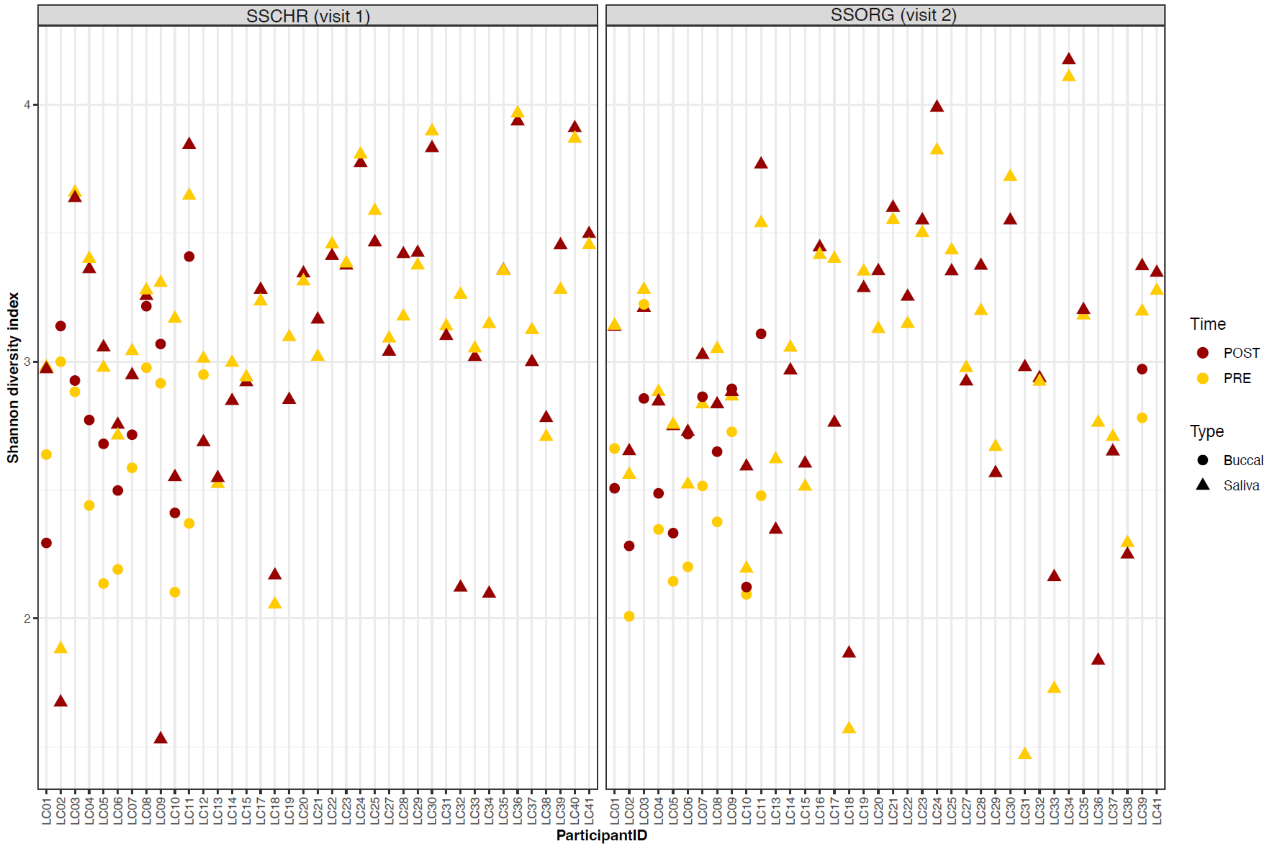

Figure S1: Alpha diversity analysis of oral bacterial microbiota per individual subject from buccal and saliva samples collected pre- (red) and post-smoking (yellow) of two separate little cigar products: Swisher sweets original (SSORG) and Swisher sweets cherry (SSCHR).

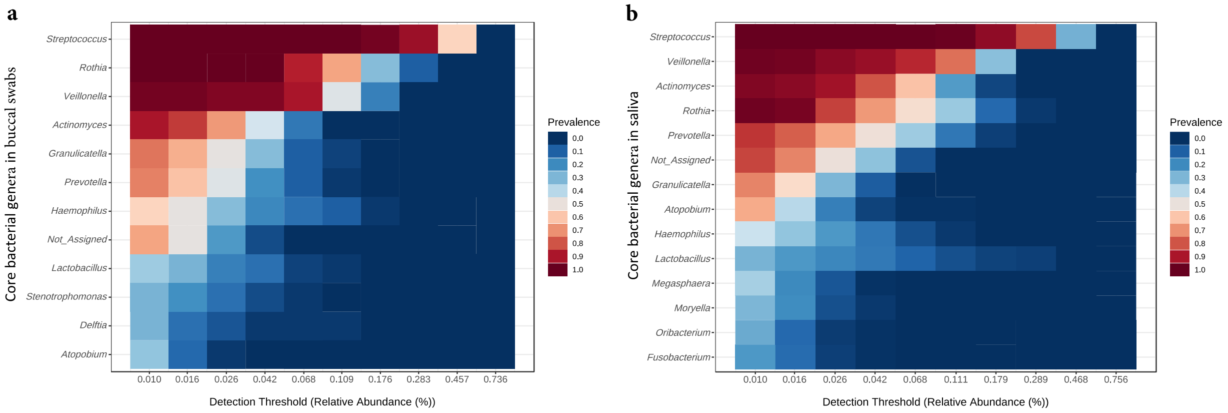

Figure S2: Core bacterial genera present in (a) buccal swab samples and (b) saliva samples. The color gradient shows the prevalence (%) of each genus from zero (blue) to all (red) samples.

Table S1: Relative abundance of top 10 bacterial genera in pre-smoking samples across all participants during two visits.

Subject ID	Days (#) between two visits	Bacterial genera									
		Actinomyces	*Atopobium*	*Granulicatella*	*Haemophilus*	*Lactobacillus*	*Prevotella*	*Pseudomonas*	*Rothia*	*Streptococcus*	*Veillonella*
LC01	14	0.136	0.037	0.003	0.000	0.155	0.059	0.007	0.119	0.956	0.307
SSCHR (visit 1)		0.100	0.020	0.001	0.000	0.058	0.027	0.005	0.050	0.505	0.112
SSORG (visit 2)		0.035	0.017	0.001	0.000	0.097	0.031	0.002	0.069	0.451	0.195
LC02	14	0.067	0.013	0.036	0.001	0.011	0.013	0.001	0.263	1.325	0.209
SSCHR (visit 1)		0.029	0.003	0.016	0.001	0.006	0.006	0.000	0.180	0.716	0.014
SSORG (visit 2)		0.038	0.010	0.020	0.001	0.005	0.007	0.001	0.083	0.609	0.195
LC03	7	0.159	0.025	0.052	0.011	0.041	0.079	0.039	0.243	0.760	0.204
SSCHR (visit 1)		0.084	0.012	0.023	0.007	0.014	0.035	0.004	0.120	0.448	0.094
SSORG (visit 2)		0.075	0.013	0.029	0.004	0.026	0.044	0.035	0.123	0.312	0.110
LC04	22	0.108	0.021	0.031	0.016	0.070	0.037	0.001	0.311	0.970	0.288
SSCHR (visit 1)		0.057	0.011	0.011	0.009	0.065	0.018	0.000	0.178	0.408	0.167
SSORG (visit 2)		0.051	0.010	0.020	0.007	0.006	0.019	0.000	0.133	0.562	0.121
LC05	12	0.090	0.012	0.062	0.112	0.008	0.048	0.002	0.208	1.115	0.232
SSCHR (visit 1)		0.032	0.004	0.039	0.088	0.005	0.028	0.001	0.113	0.528	0.098
SSORG (visit 2)		0.058	0.008	0.023	0.023	0.003	0.021	0.001	0.095	0.588	0.134
LC06	5	0.109	0.017	0.117	0.006	0.003	0.051	0.001	0.377	0.917	0.317
SSCHR (visit 1)		0.063	0.011	0.047	0.003	0.001	0.034	0.000	0.153	0.459	0.179
SSORG (visit 2)		0.046	0.006	0.070	0.003	0.002	0.017	0.000	0.224	0.458	0.138
LC07	4	0.057	0.027	0.037	0.004	0.082	0.100	0.002	0.180	0.948	0.391
SSCHR (visit 1)		0.029	0.013	0.026	0.003	0.041	0.040	0.002	0.128	0.473	0.158
SSORG (visit 2)		0.028	0.014	0.010	0.001	0.041	0.060	0.000	0.052	0.474	0.233
LC08	2	0.145	0.024	0.070	0.056	0.005	0.156	0.001	0.175	0.872	0.334
SSCHR (visit 1)		0.084	0.014	0.041	0.037	0.001	0.102	0.000	0.072	0.359	0.175
SSORG (visit 2)		0.061	0.010	0.029	0.019	0.004	0.054	0.000	0.102	0.514	0.159
LC09	4	0.150	0.012	0.093	0.040	0.004	0.078	0.077	0.208	0.825	0.362
SSCHR (visit 1)		0.075	0.004	0.064	0.019	0.001	0.058	0.001	0.102	0.414	0.178
SSORG (visit 2)		0.075	0.008	0.029	0.021	0.003	0.020	0.076	0.107	0.411	0.184
LC10	2	0.123	0.007	0.023	0.224	0.008	0.023	0.002	0.379	0.876	0.206
SSCHR (visit 1)		0.079	0.005	0.014	0.108	0.003	0.019	0.001	0.173	0.371	0.141
SSORG (visit 2)		0.043	0.002	0.009	0.116	0.005	0.004	0.001	0.205	0.505	0.065
LC11	5	0.231	0.018	0.016	0.071	0.003	0.099	0.002	0.471	0.639	0.169
SSCHR (visit 1)		0.107	0.007	0.007	0.048	0.002	0.040	0.002	0.238	0.308	0.070

SSORG (visit 2)		0.124	0.011	0.008	0.023	0.001	0.059	0.000	0.232	0.330	0.100
LC12	26	0.019	0.006	0.005	0.001	0.299	0.014	0.009	0.132	0.284	0.078
SSCHR (visit 1)		0.019	0.006	0.005	0.001	0.299	0.014	0.009	0.132	0.284	0.078
LC13	4	0.112	0.016	0.045	0.002	0.026	0.030	0.004	0.151	1.259	0.159
SSCHR (visit 1)		0.061	0.012	0.017	0.001	0.018	0.025	0.001	0.067	0.595	0.118
SSORG (visit 2)		0.051	0.003	0.028	0.000	0.008	0.006	0.003	0.085	0.664	0.041
LC14	5	0.222	0.005	0.122	0.056	0.010	0.020	0.000	0.234	0.923	0.159
SSCHR (visit 1)		0.100	0.001	0.071	0.034	0.007	0.005	0.000	0.151	0.466	0.015
SSORG (visit 2)		0.121	0.004	0.050	0.023	0.003	0.015	0.000	0.083	0.457	0.144
LC15	1	0.145	0.006	0.107	0.072	0.013	0.094	0.000	0.264	0.854	0.295
SSCHR (visit 1)		0.076	0.003	0.067	0.066	0.004	0.070	0.000	0.099	0.377	0.127
SSORG (visit 2)		0.069	0.003	0.040	0.006	0.009	0.025	0.000	0.164	0.477	0.168
LC16	4	0.073	0.020	0.014	0.006	0.004	0.061	0.000	0.008	0.442	0.154
SSORG (visit 2)		0.073	0.020	0.014	0.006	0.004	0.061	0.000	0.008	0.442	0.154
LC17	6	0.105	0.011	0.027	0.019	0.001	0.106	0.000	0.037	0.389	0.194
SSCHR (visit 1)		0.105	0.011	0.027	0.019	0.001	0.106	0.000	0.037	0.389	0.194
LC18	5	0.011	0.004	0.002	0.003	0.114	0.007	0.001	0.617	1.172	0.040
SSCHR (visit 1)		0.009	0.002	0.002	0.002	0.096	0.005	0.000	0.343	0.507	0.017
SSORG (visit 2)		0.002	0.002	0.000	0.001	0.018	0.002	0.001	0.274	0.664	0.023
LC19	7	0.198	0.010	0.010	0.035	0.008	0.050	0.000	0.131	0.906	0.292
SSCHR (visit 1)		0.101	0.006	0.007	0.024	0.003	0.035	0.000	0.055	0.438	0.183
SSORG (visit 2)		0.098	0.004	0.003	0.011	0.006	0.014	0.000	0.075	0.468	0.109
LC20	2	0.132	0.045	0.072	0.043	0.015	0.167	0.000	0.236	0.778	0.327
SSCHR (visit 1)		0.066	0.026	0.029	0.037	0.007	0.093	0.000	0.132	0.362	0.140
SSORG (visit 2)		0.066	0.019	0.043	0.006	0.008	0.075	0.000	0.104	0.417	0.187
LC21	4	0.223	0.022	0.032	0.014	0.007	0.270	0.000	0.048	0.722	0.326
SSCHR (visit 1)		0.070	0.011	0.016	0.006	0.004	0.138	0.000	0.024	0.430	0.199
SSORG (visit 2)		0.154	0.011	0.017	0.008	0.002	0.132	0.000	0.024	0.292	0.126
LC22	7	0.166	0.038	0.028	0.054	0.004	0.186	0.000	0.080	0.770	0.398
SSCHR (visit 1)		0.090	0.029	0.007	0.010	0.004	0.115	0.000	0.017	0.357	0.197
SSORG (visit 2)		0.076	0.008	0.022	0.043	0.001	0.071	0.000	0.063	0.413	0.201
LC23	2	0.193	0.038	0.007	0.005	0.006	0.183	0.001	0.039	0.711	0.375
SSCHR (visit 1)		0.083	0.019	0.003	0.003	0.005	0.119	0.000	0.016	0.333	0.208
SSORG (visit 2)		0.109	0.019	0.004	0.003	0.001	0.064	0.000	0.023	0.378	0.167
LC24	6	0.193	0.053	0.026	0.015	0.003	0.331	0.000	0.054	0.412	0.366
SSCHR (visit 1)		0.102	0.028	0.013	0.007	0.002	0.159	0.000	0.026	0.217	0.180
SSORG (visit 2)		0.090	0.024	0.013	0.008	0.001	0.172	0.000	0.028	0.195	0.186
LC25	5	0.347	0.031	0.023	0.014	0.005	0.153	0.001	0.037	0.649	0.352

SSCHR (visit 1)		0.155	0.015	0.014	0.013	0.002	0.102	0.000	0.019	0.317	0.164
SSORG (visit 2)		0.191	0.016	0.009	0.001	0.003	0.050	0.000	0.018	0.332	0.188
LC27	5	0.262	0.083	0.027	0.000	0.183	0.106	0.001	0.134	0.545	0.471
SSCHR (visit 1)		0.189	0.048	0.013	0.000	0.053	0.043	0.001	0.039	0.203	0.269
SSORG (visit 2)		0.073	0.034	0.014	0.000	0.130	0.063	0.000	0.095	0.342	0.201
LC28	2	0.299	0.047	0.021	0.006	0.012	0.201	0.001	0.054	0.615	0.459
SSCHR (visit 1)		0.133	0.025	0.013	0.003	0.003	0.139	0.001	0.024	0.343	0.175
SSORG (visit 2)		0.166	0.022	0.008	0.004	0.010	0.062	0.001	0.030	0.272	0.284
LC29	7	0.134	0.011	0.005	0.030	0.006	0.055	0.000	0.044	0.456	0.109
SSCHR (visit 1)		0.134	0.011	0.005	0.030	0.006	0.055	0.000	0.044	0.456	0.109
LC30	6	0.172	0.030	0.030	0.039	0.004	0.145	0.001	0.068	0.693	0.300
SSCHR (visit 1)		0.061	0.013	0.017	0.027	0.001	0.075	0.000	0.046	0.332	0.142
SSORG (visit 2)		0.111	0.016	0.013	0.012	0.003	0.070	0.000	0.022	0.361	0.158
LC31	2	0.039	0.013	0.051	0.004	0.066	0.043	0.690	0.126	0.668	0.163
SSCHR (visit 1)		0.033	0.011	0.049	0.003	0.050	0.040	0.000	0.106	0.460	0.157
SSORG (visit 2)		0.006	0.002	0.001	0.001	0.016	0.003	0.690	0.020	0.208	0.006
LC32	2	0.086	0.008	0.029	0.051	0.007	0.082	0.000	0.186	0.322	0.130
SSORG (visit 2)		0.086	0.008	0.029	0.051	0.007	0.082	0.000	0.186	0.322	0.130
LC33	3	0.072	0.009	0.026	0.027	0.004	0.028	0.538	0.135	0.675	0.191
SSCHR (visit 1)		0.062	0.006	0.023	0.024	0.002	0.027	0.001	0.126	0.442	0.158
SSORG (visit 2)		0.010	0.003	0.003	0.003	0.001	0.001	0.537	0.009	0.233	0.033
LC34	17	0.139	0.047	0.024	0.028	0.008	0.148	0.346	0.020	0.419	0.203
SSCHR (visit 1)		0.068	0.033	0.009	0.006	0.004	0.035	0.345	0.004	0.154	0.109
SSORG (visit 2)		0.071	0.014	0.015	0.022	0.004	0.113	0.001	0.016	0.265	0.094
LC35	7	0.154	0.015	0.047	0.191	0.008	0.069	0.000	0.143	0.786	0.198
SSCHR (visit 1)		0.066	0.007	0.025	0.070	0.005	0.059	0.000	0.063	0.406	0.097
SSORG (visit 2)		0.088	0.008	0.023	0.121	0.002	0.011	0.000	0.080	0.380	0.101
LC36	35	0.112	0.031	0.014	0.030	0.003	0.147	0.004	0.021	0.149	0.154
SSCHR (visit 1)		0.112	0.031	0.014	0.030	0.003	0.147	0.004	0.021	0.149	0.154
LC37	2	0.102	0.015	0.074	0.066	0.011	0.099	0.000	0.340	0.806	0.324
SSCHR (visit 1)		0.068	0.009	0.051	0.066	0.003	0.081	0.000	0.152	0.328	0.153
SSORG (visit 2)		0.034	0.006	0.023	0.001	0.008	0.018	0.000	0.187	0.478	0.171
LC38	10	0.116	0.016	0.086	0.000	0.046	0.100	0.000	0.399	0.926	0.244
SSCHR (visit 1)		0.080	0.011	0.045	0.000	0.028	0.094	0.000	0.131	0.374	0.194
SSORG (visit 2)		0.036	0.005	0.040	0.000	0.019	0.006	0.000	0.268	0.552	0.050
LC39	2	0.206	0.024	0.110	0.017	0.012	0.286	0.032	0.057	0.472	0.508
SSCHR (visit 1)		0.091	0.013	0.028	0.004	0.011	0.176	0.001	0.016	0.214	0.262

SSORG (visit 2)		0.115	0.011	0.082	0.013	0.001	0.110	0.031	0.041	0.258	0.246
LC40	6	0.073	0.008	0.023	0.039	0.003	0.113	0.000	0.011	0.210	0.126
SSCHR (visit 1)		0.073	0.008	0.023	0.039	0.003	0.113	0.000	0.011	0.210	0.126
LC41	4	0.218	0.028	0.045	0.108	0.012	0.102	0.001	0.257	0.579	0.365
SSCHR (visit 1)		0.108	0.013	0.020	0.046	0.005	0.063	0.001	0.121	0.293	0.174
SSORG (visit 2)		0.110	0.015	0.025	0.062	0.007	0.039	0.000	0.136	0.287	0.191
